# Supplementary material for: MaqFACS (Macaque Facial Action Coding System) can be used to document facial movements in Barbary macaques (Macaca sylvanus)
Source: PeerJ. 2015 Sep 15;3:e1248. doi: 10.7717/peerj.1248 (PMC4579026; doi:10.7717/peerj.1248)
Supplement: Supplemental Information 10 [file peerj-03-1248-s010.docx]

**Supplementary Material**

Videos illustrating the different AUs (please read using VLC media player)

For each video, please refer to the time code indicated (i.e. 00:06).

SupplementalVideoS01 – AU1+2 (00:02)

Movement upward of the browline, revealing a greater surface area in the underbrow region

SupplementalVideoS02 – AU1+2 + EAU2 (00:06)

Browline and ears move upward, pulling the ears towards the top of the head

SupplementalVideoS03 – AU41 + EAU3 (00:05)

Lowering of the browline reducing the visibility of the underbrow region

SupplementalVideoS04 – AU41 + AU9+10 + AU16 + AU25 + AU27 (00:03)

Here the individual yawns, using AUs of the upper (lowering the browline) and lower face (mouth opening, upper lip pulled upward with AU9+10/AU10 and lower lip pulled downward with AU16)

SupplementalVideoS05 – AU12 (00:02)

The corner of the mouth is pulled back and slightly upward under the action of AU12

SupplementalVideoS06– AU1+2 + AU18i + AU25 + AU26 + EAU1 (00:02)

In this video, the browline is slightly pulled upward (AU1+2), the corners of the mouth are pulled medially reducing the mouth aperture (AU18i) and the lips and jaws are parted (AU25 + AU26). The ears can be seen moving forwards (EAU1), reducing the ear surface visible

SupplementalVideoS07 – AU1+2 + AU18ii + AU25 + AU26 (00:08 for best visibility)

Here we can see the second type of lips protrusion, where the lips and the lower jaw are pushed forward so as to protrude slightly. Moreover, we can see the browline moving upward.

SupplementalVideoS08 – AU1+2 + EAU1 (00:03)

The browline moves upward and the ears move forwards on this video.
